# Evaluation of LC-MS and LC×LC-MS in analysis of zebrafish embryo samples for comprehensive lipid profiling

**DOI:** 10.1007/s00216-020-02661-1

**Published:** 2020-04-29

**Authors:** Mengmeng Xu, Jessica Legradi, Pim Leonards

**Affiliations:** Department of Environment and Health, Vrije Universiteit, De Boelelaan 1085, 1081 HV Amsterdam, The Netherlands

**Keywords:** Comprehensive two-dimensional liquid chromatography, Conventional one-dimensional liquid chromatography, Untargeted lipidomics, Zebrafish

## Abstract

In this study, both conventional one-dimensional liquid chromatography (1DLC) and comprehensive two-dimensional liquid chromatography (2DLC) coupled to a high-resolution time-of-flight mass spectrometer (HR-TOF MS) were used for full-scale lipid characterization of lipid extracts from zebrafish embryos. We investigated the influence on annotated lipids and different separation mechanisms (HILIC, C18, and PFP), and their different orders arranged in the first and the second dimensions. As a result, the number of lipid species annotated by conventional one-dimensional LC-MS was between 212 and 448. In contrast, the number of individual lipids species annotated by C18×HILIC, HILIC×C18, and HILIC×PFP were 1784, 1059, and 1123, respectively. Therefore, it was evident that the performance of comprehensive 2DLC, especially the C18×HILIC method, considerably exceeded 1DLC. Interestingly, a comparison of the HILIC×C18 and C18×HILIC approaches showed, under the optimized conditions, similar orthogonality, but the effective separation power of the C18×HILIC was much higher. A comparison of the HILIC×C18 and the HILIC×PFP methods demonstrated that the HILIC×PFP separation had superior orthogonality with a small increase on its effective peak capacity, indicating that the HILIC×PFP combination maybe a promising platform for untargeted lipidomics in complex samples. Finally, from the comprehensive lipid profiling respective, the C18×HILIC was selected for further studies.

**Electronic supplementary material:**

The online version of this article (10.1007/s00216-020-02661-1) contains supplementary material, which is available to authorized users.

## Introduction

Lipidomics is a fast-growing field, which aims to study the full spectrum of lipid species and the interaction among lipids in biological system. Lipids play an essential role in energy storage, structural composition and cell signaling. The link between lipids and cancer, kidney diseases, diabetes, cardiovascular diseases, neurological disorders, obesity, and depression or anxiety has been previously reviewed [1–6]. Besides, it has been reported that a loss in homeostasis of lipids can be related to exposure of external environmental factors, such as exposure to metals [7] and pesticides [8]. A better understanding of lipid profiling in biological fluids (e.g., urine, blood, and tissue samples) offers also a cornerstone to raise the possibility for the discovery of potential biomarkers or new mechanisms related to diseases [9, 10]. Therefore, comprehensive analysis of lipids is gaining more attention in many fields, ranging from biomedical to environmental studies.

The objective in untargeted lipidomics is to obtain as much information and characterization in lipid components as possible. Until now, liquid chromatography (LC) coupled to high-resolution mass spectrometry (HRMS) is most frequently applied to separate the high diversity of lipid species before injected into the MS [11–13]. Further, various advanced techniques have been developed to study the lipidome, using different mass spectrometry strategies. The combination of nanoflow LC and trapped ion mobility spectrometry (TIMS), for example, enabled high-throughput lipidomics analysis, even with a limited amount of sample [14]. The structure specificity of individual lipids, especially the C=C location and sn-position, could be qualitatively and quantitatively identified by coupling photochemical (Paternò-Bǜchi, PB) reaction with tandem MS (MS/MS) [15, 16]. The versatility of LC is extremely successful because of many different separation mechanism such as reverse-phase (RP), normal-phase (NP), and hydrophilic interaction (HILIC) LC. RPLC (e.g., C18, C8 [12], and C30 [17]) as stationary phases provides a good resolution of lipids in separation based on hydrophobicity which consists of the length of acyl chains, and the number and position of double bond (DB) [18]. It has emerged as a state-of-the-art technique because of its broad coverage of analytes, from nonpolar to polar lipids. Apart from the RPLC mechanisms described above, little was known about RPLC columns with other stationary phases, such as phenyl-hexyl and pentafluorophenyl (PFP), which are, to some content, different from the prevailing phases because a PFP-embedded column, for example, has multiple separation mechanisms (e.g., hydrophobic, π-π interaction, dipole-dipole, H bonding, and shape selectivity), while a C18 column separates mainly on the hydrophobicity. It shows a limitation on the diversity of RPLC modes applied to untargeted lipidomics, and also the possibilities to discover potential modes for good separation in LC-MS analysis. Recently, HILIC as an alternative technique to NPLC is increasingly used to determinate individual lipid classes using a mobile phase system which is more friendly and compatible to the MS detection [19, 20].

Among two-dimensional liquid chromatography (2DLC) systems, there are a variety of offline and online configurations which have both strengths and weaknesses. Online comprehensive two-dimensional LC (LC×LC)-MS has a higher peak capacity under ideal conditions when the two separation modes are completely orthogonal and the total peak capacity of the approaches is equal to the product of peak capacities in the first dimension and the second dimension, while it can also be automated. Another attractive advantage of LC×LC-MS is that this technique provides more information on the identity of the lipid species because chromatographic behaviors are different between the two dimensions.

The strengths of the 2DLC analysis compared with 1DLC have been discussed in literatures [21, 22], but the real-world performance of these two types of analytical techniques for comprehensive lipid profiling for zebrafish (*Danio rerio*) has not been compared in detail. Zebrafish are used as model organisms in many different areas such as developmental biology, as a genetic model system in testing chemicals for toxicity, in human toxicology, and in drug discovery. In general, a limited number of papers have focused on the evaluation and optimization of analytical methods to determine targeted analyses of lipids, such as prenol lipids [23] and fatty acids [24] in zebrafish. Only a few articles focused on comprehensive lipid profiling of zebrafish and used conventional LC-MS methods [25, 26]. To our knowledge, this is the first study on the development and application of 2DLC-MS lipidomics approaches in zebrafish. However, 2DLC-MS lipidomics has been used in other fields such as human plasma [27–30] and plants [31, 32]. The aim of this study was to optimize a variety of LC×LC-MS methods based on C18×HILIC, HILIC×C18, and HILIC×PFP, using the lipid classes found in zebrafish embryos. Simultaneously, the same samples were analyzed by conventional 1DLC-MS using C18, PFP, and HILIC phases.

The performance of different LC×LC-MS methods, which have inverse orders of two dimensions (C18×HILIC vs HILIC×C18), or different selectivities in the second dimensions (HILIC×C18 vs HILIC×PFP) were then accessed by the number of lipid annotations, the orthogonality, and the effective peak capacity. To the best of our knowledge, only a few studies have compared the separation space and power of 1DLC-MS and 2DLC-MS with lipids from biological samples.

## Materials and methods

### Chemicals and compounds

Milli-Q water was produced from a Millipore purification system (Waters-Millipore Corporation, Milford, MA, USA). HPLC grade acetonitrile (ACN), 2-isopanol (IPA), chloroform, and methanol (MeOH) were obtained from JT Baker Chemical (Phillipsburg, NJ, USA). MS grade formic acid (98% purity) and ammonium formate salt (≥ 99% purity) were from Fluka (Steinheim, Germany).

For the preliminary optimization of methods, fourteen lipid standards were mixed and used in untargeted lipidomics studies (Table 1). In addition, internal isotope-labelled standards (SPLASH LIPIDOMIX Mass Spec Standard) which contains major lipid classes were used (see Table S5 in the Electronic Supplementary Material, ESM). All lipid standards and internal standards were purchased from Avanti Polar Lipids (Alabaster, AL, USA).

**Table 1:** List of lipid standards and their structural as well as mass spectrometry information

Lipid class	Abbreviation	Acyl chains	m/z	Adduct
Diacylglycerol	DG	(16:0/18:1)	612.5	[M+NH4]^+^
		(18:1/18:1)	638.5	[M+NH4]^+^
Triacylglycerol	TG	(18:1/18:1/18:1)	902.8	[M+NH4]^+^
Ceramide	CER	(18:1/18:1)	564.5	[M+H]^+^
		(18:0/18:1)	566.5	[M+H]^+^
Sphingomyeline	SM	(18:0/18:0)	731.6	[M+H]^+^
Phosphatidylcholine	PC	(18:1/18:1)	786.6	[M+H]^+^
		(18:0/18:0)	790.6	[M+H]^+^
Phosphatidylinositol	PI	(18:1/18:1)	863.5	[M+H]^+^
		(18:0/20:4)	887.5	[M+H]^+^
Phosphatidic acid	PA	(18:1/18:1)	700.5	[M+H]^+^
Phosphatidylglycerol	PG	(18:1/18:1)	775.5	[M+H]^+^
Phosphatidylethanolamine	PE	(18:1/18:1)	744.5	[M+H]^+^
Phosphatidylserine	PS	(18:1/18:1)	788.5	[M+H]^+^

The stock solutions of each standard (about 1000 mg L^−1^) were separately prepared in chloroform-methanol (2:1, v/v). The working solution of all standards (10 mg mL^−1^) was prepared by evaporation of initial solvents and re-dissolving the dried samples in IPA/ACN/H_2_O (5:4:1, v/v/v).

### Lipid extraction of zebrafish embryos

Wild-type zebrafish obtained from Ruinemans (Montfoort, The Netherlands) were kept in our animal facility under standard conditions. Adult fish were separated overnight in a breeding cage to induce spawning the next morning. Within 1 h post fertilization, eggs were collected and transferred into a petri dish filled with Embryo Standard Water (ESW; 100 mgL^−1^ NaHCO_3_, 20 mgL^−1^ KHCO_3_, 180 mgL^−1^ MgSO_4_, and 200 mgL^−1^ CaCl_2_) at 26 °C. The quality of the eggs was assessed daily under a stereo microscope (M7.5, Leica, Eindhoven, The Netherlands). Six batches of fifteen zebrafish embryos of 5 day post fertilization were collected without water in 2-mL Precellys tubes with 1.4-mm ceramic beads (zirconium oxide) (CK 14, Bertin Technologies, France) and snap frozen and stored at − 80 °C. According to Directive 2010/63/EU, no ethical approval was needed for this study.

The lipid extraction was performed by a Precellys 24 Dual device (Bertin Technologies, France), operating at 6500 rpm for two cycles of 10 s with a 15-s break between cycles. The first and second homogenization were carried out with 150 μL of H_2_O (ice cold) and 150 μL of methanol (− 80 °C cold), respectively. For the third homogenization, 290 μL of chloroform was added into samples with 10 μL internal standard solution. In order to enhance the efficiency of protein precipitation, partitioning of 10 min is necessary in ice before centrifuging of 5 min at 4 °C operating at 15,000 rpm. Finally, 100 μL of the bottom organic layer was collected and evaporated under a gentle stream of nitrogen. Dried lipid extracts were dissolved using 100 μL of the mixture of ACN/IPA/H_2_O (5:4:1, v/v/v), and stored at – 80 °C prior to chromatographic analysis.

### Conventional 1DLC

The 1DLC system consisted of an Agilent 1200 HPLC system (Agilent, Palo Alto, USA) coupled with a high-resolution time-of-flight MS (Micro TOF, Bruker Daltonik, Bremen, Germany). The HPLC system consisted of a quaternary pump, a vacuum degasser, an autosampler with a cooling unit (4 °C), and a heated column compartment.

Two different RPLC modes for untargeted lipidomics were used. Firstly, the chromatographic separation of lipid extracts was achieved using a Kinetex EVO C18 column (100 × 2.1 mm, 2.6 μm particle size; Phenomenex, USA). The mobile phase consisted of A1 acetonitrile in water (60:40, v/v) with ammonium formate (10 mM) and formic acid (0.1%), and B1 isopropanol in acetonitrile (90:10, v/v) with ammonium formate (10 mM) and formic acid (0.1%). The gradient elution was as follows: 0 min 15% (B1); 0–2 min 30% (B1); 2–3 min 48% (B1); 3–20 min 82% (B1); 20–21 min 99% (B1); 21–30 min 99% (B1); 30–32 min 15% (B1). Secondly, a Kinetex pentafluorophenyl column (PFP, 50 × 4.6 mm, 2.6 μm particle size; Phenomenex, USA) was applied using the same mobile phase solvents as the C18 column. The gradient was as follows: 0 min 20% (B1); 0–25 min 99% (B1); 25–28 min 99% (B1); 28–31 min 20% (B1). The two columns were maintained using identical parameters, which included injection volume of 5 μL, column temperature of 45 °C, and flow rate of 0.25 mL min^−1^.

In addition, a HILIC XBridge Amide column (150 × 2.1 mm, 3.5 μm particle size; Waters, USA) was used for the separation of the lipids using the following conditions: injection volume of 5 μL; column temperature of 45 °C; flow rate of 0.20 mL min^−1^; gradient slope was shown: 0 min 100% (B2); 0–10 min 80% (B2); 10–25 min 20% (B2); 25–28 min 100% (B2) where mobile phase A2 consisted of 50% water 50% acetonitrile, and mobile phase B2 of 5% water 95% acetonitrile, both of them contained 0.1% formic acid and 10 mM ammonium formate.

### Comprehensive 2DLC

The two-dimensional liquid chromatography system consisted of an Agilent 1100 auto sampler (G1330A), an Agilent 1290 infinity thermostatted column compartment (G1316C), an Agilent 1100 HPLC binary pump (G1312A) for the first dimension, and an Agilent 1290 infinity UHPLC binary pump (G4220B) for the second dimension. A two-position/four-port duo valve (Agilent Technologies, Waldbronn, Germany) with two sampling loops (40 μL) was installed as the 2D interface. The 2DLC system was operated by Openlab CDS Chemstation (reversion C.01.07) with 2D-LC add-on software (reversion B.04.03). The LC×LC methods were developed based on the combinations of three mechanisms (C18, HILIC, and PFP), which were shown in the form of C18×HILIC (a), HILIC×C18 (b), and HILIC×PFP (c). Combination A consisted of an EVO C18 column (100 × 2.1 mm, 2.6 μm) and a BEH HILIC column (50 × 2.1 mm, 1.7 μm; Waters, USA). The combination of a XBridge Amide column (150 × 2.1 mm, 3.5 μm) and a Tina C18 column (50 × 3.0 mm, 1.9 μm, Sigma-Aldrich, USA) and the combination of the same amide column and a Kinetex PFP column (50 × 4.6 mm, 2.6 μm) were selected for combinations B and C, respectively. The ^2^D effluent was split by a QuickSplit™ adjustable flow splitter (Richmond, CA, USA): 20% was transferred into the MS system, 80% was directed into waste. The chromatographic conditions of the LC×LC methods are listed in Table 2 and the optimal conditions used for the untargeted lipidomic analysis of zebrafish samples as well.

**Table 2:** The conditions of the C18×HILC, HILIC×18, and HILIC×PFP approaches

Column	First dimensional LC conditions	Second dimensional LC conditions
C18×HILIC	Mobile phase (A): acetonitrile:water (60:40, v/v), (B): 2-isopronol:acetonitrile (90:10, v/v), both contain 10 mM HCOONH_4_ Gradient: 0 min 40% B, 139 min 99% B, 155 min 99% B, 155.1 min 40% B, 170 min 40% B Flow rate: 20 μL min^−1^ Temperature: 55 °C	Mobile phase (A): water, (B): acetonitrile:water (95:5, v/v), both contain 10 mM HCOONH_4_ Full gradient: 0 min 95% B, 0.8 min 86.5% B, 0.81 min 95% B, 1 min 95% B Flow rate: 2 mL min^−1^ Temperature: 40 °C
HILIC×C18	Mobile phase (A): acetonitrile:water (50:50, v/v), (B): acetonitrile:water (95:5, v/v), both contain 10 mM HCOONH_4_ and 0.1% HCOOH Gradient: 0 min 95% B, 30 min 82% B, 50 min 82% B, 90 min 65% B, 90.1 min 95% B, 100 min 95% B Flow rate: 20 μL min^−1^ Temperature: 45 °C	Mobile phase (A): acetonitrile:water (60:40, v/v), (B): 2-isopronol:acetonitrile (90:10, v/v), both contain 10 mM HCOONH_4_ and 0.1% HCOOH Segment gradients: (1) 0–40 min: 0 min 40% B, 1.45 min 99% B, 1.85 min 99% B, 1.86 min 40% B, 2 min 40% B; (2) 40–60 min: 0 min 50% B, 1.85 min 99% B, 1.86 min 50% B, 2 min 50% B; (3) 60–90 min: 0 min 20% B, 1.85 min 65% B, 1.86 min 20% B, 2 min 20% B Flow rate: 1.5 mL min^−1^ Temperature: 60 °C
HILIC×PFP	Mobile phase (A): acetonitrile:water (50:50, v/v), (B): acetonitrile:water (95:5, v/v), both contain 10 mM HCOONH_4_ and 0.1% HCOOH Gradient: 0 min 95% B, 30 min 82% B, 50 min 82% B, 90 min 65% B, 90.1 min 95% B, 100 min 95% B Flow rate: 20 μL min^−1^ Temperature: 45 °C	Mobile phase (A): acetonitrile:water (60:40, v/v), (B): 2-isopronol:acetonitrile (90:10, v/v), both contain 10 mM HCOONH_4_ and 0.1% HCOOH Segment gradients: (1) 0–40 min: 0 min 20% B, 1.45 min 99% B, 1.85 min 99% B, 1.86 min 20% B, 2 min 20% B; (2) 40–60 min: 0 min 45% B, 1.35 min 99% B, 1.85 min 99% B, 1.86 min 45% B, 2 min 45% B; (3) 60–90 min: 0 min 20% B, 1.85 min 65% B, 1.86 min 20% B, 2 min 20% B Flow rate: 2 mL min^−1^ Temperature: 50 °C

### MS conditions

A Bruker micrOTOF™ time-of-flight (TOF) mass spectrometer equipped with an electrospray ionization (ESI) source (Bruker Daltonics, Bremen, Germany) was used for the detection of the lipids in both positive and negative modes. The MS parameter settings were as follows: capillary voltages of ± 4500 V, end set plates of ± 500 V, nebulizer gas (N_2_) pressure of 2 bar, drying gas flow rate of 6 L min^−1^, and the drying gas temperature was 250 °C. The m/z detection of MS was determined in the range from 50 to 1500 in positive mode with the sampling rate of 6 Hz. The Q-TOF was controlled by Bruker qtof Control version 3.0.

### Data processing and visualization

Raw MS data were initially processed using instrument software packages DataAnalysis (version 4.1, Bruker Daltonics). The total ion chromatograms (TICs) obtained were first calibrated internally by creating a calibration segment prior to the analysis using the calibration tune mix solution on high-precision calibration (HPC) in DataAnalysis.

For visualization of the LC×LC data, calibrated chromatographic data was imported in GC Image (University of Nebraska, Lincoln, NE, USA) after being converted to a netCDF file by the DataAnalysis software. The chromatographic data was transferred into MZXL format which was then processed in MSDial [33] (v. 3.70) for annotation of the lipids from zebrafish extracts. The important parameters of this software were set as follows: retention time begin, 0.3 min; retention time end, 170 min; mass range begin, 60 Da; mass range end, 1500 Da; MS1 (centroiding) tolerance, 0.005 Da; MS2 (centroiding) tolerance, 0.01 Da; smoothing level, 3 scans; minimum peak height, 500 amplitude; mass slice width, 0.05 Da; accurate mass tolerance (MS1), 0.02 Da; accurate mass tolerance (MS2), 0.02 Da; identification score cutoff, 70%; without using retention information for scoring.

Excel software (Microsoft, WA, USA) was used to calculate the orthogonality of the LC×LC system. Besides, peak capacity of 2DLC systems is the theoretical peak number under the given conditions, which is one of the essential parameters for separation power. According to the findings supported by Li et al. [34], the approximate equation model of the corrected peak capacity, including under-sampling, was given as:

1 $$ {n}_{\mathrm{c},2\mathrm{D}}\cong \frac{{1_t}_{\mathrm{g}}{2_n}_{\mathrm{c}}}{1.83\ {2_t}_{\mathrm{c}}} $$

where ^1^*t*_g_ is the gradient time of the first dimension, ^2^*n*_c_ is the peak capacity of the second dimension, and ^2^*t*_c_ is the cycle time of the second dimension.

Furthermore, if we consider the influence of the orthogonality on the separation space of the LC×LC methods, the equation of the effective peak capacity (practical peak capacity) comprehensive 2DLC systems could be shown as:

2 $$ {n}_{\mathrm{c},2\mathrm{D}}^{\ast}\cong \frac{{1_t}_{\mathrm{g}}{2_n}_{\mathrm{c}}}{1.83\ {2_t}_{\mathrm{c}}}\times {f}_{\mathrm{c}\mathrm{overage}} $$

where *f*_coverage_ is the value of surface coverage which is between 0 and 1.

## Results and discussion

### Conventional 1DLC-MS separation

A mixture of fourteen lipid standards was used for the initial optimization of the conventional LC-MS approaches (for abbreviations and details, see Table 1, and for abbreviations of other lipid classes, see ESM). The most abundant adducts of lipid components from PC, PE, PS, SM, and CER groups were found as protonated [M+H]^+^ ions, while ions of DG, TG, and PI were observed as [M+NH_4_]^+^ adducts with a small part of [M+Na]^+^, and the adduct intensity of cholesterol was lower and shown mainly as [M+NH_4_]^+^ in real samples (see ESM Tables S1 to S4).

**Fig. 1:**
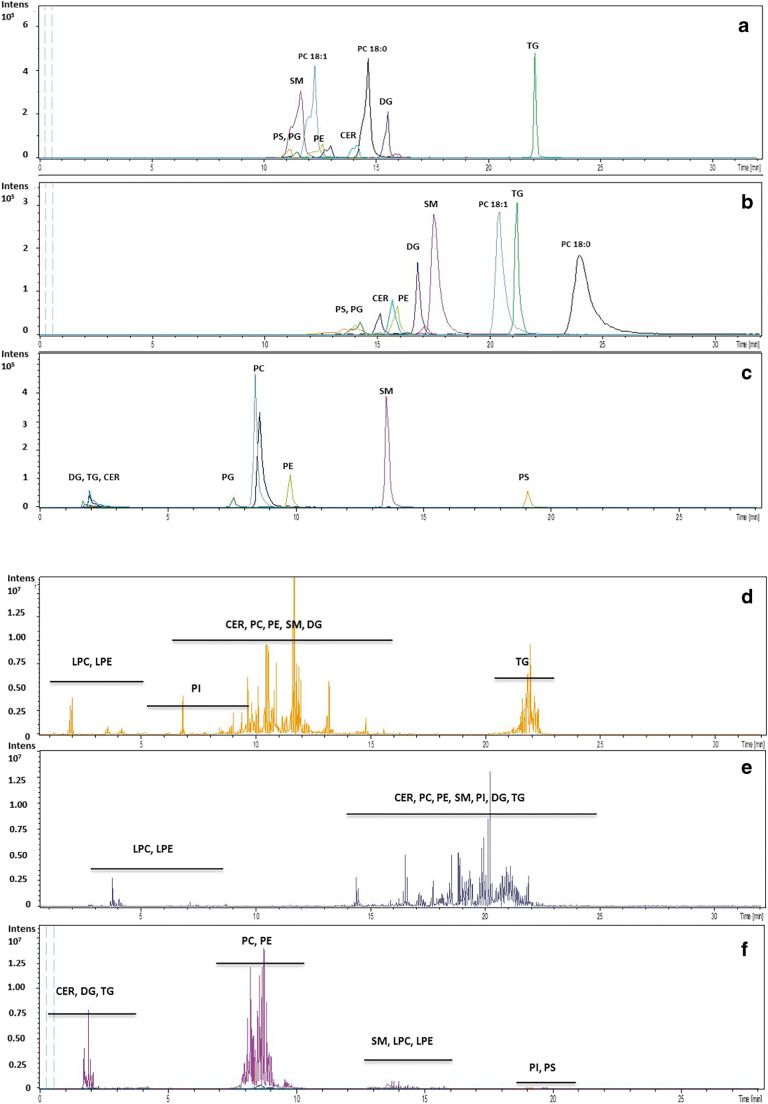
The conventional LC-MS analysis of the mixture of lipid standards by C18 (**a**), PFP (**b**), and HILIC (**c**) (extracted ion chromatograms), and the lipid extracts of zebrafish embryo samples by C18 (**d**), PFP (**e**), and HILIC (**f**) (total ion chromatograms) in positive ionization mode. The chromatographic and MS conditions are described in the “Materials and methods” section. Abbreviations of main lipid classes are as follows: lysophosphatidylcholine (LPC), phosphatidylinositol (PI), phosphatidylserine (PS), phosphatidylglycerol (PG), phosphatidylcholine (PC), phosphatidylethanolamine (PE), diacylglycerol (DG), sphingomyeline (SM), ceramide (CER), triacylglycerol (TG)

The overlaid extracted ion chromatograms of 1DLC-MS of the mixture of lipid standards are shown as Fig. 1a–c, and the total ion current chromatograms of lipid extracts from zebrafish embryos under the same conditions are shown in Fig. 1d–f.

For the C18 analysis, the retention time increased according to the increasing order of hydrophobicity of lipids (Fig. 1a), which is in agreement with results supported by Ovčačíková et al. [18]. Interestingly, for a PFP column, the retention time of PC (18:0/18:0) is even longer than that of TG (18:1/18:1/18:1) (Fig. 1b) while the TGs are eluted lastly at a C18 column. In this study, the separation modes of PFP and C18 are, to some content, orthogonal at least for PC species since a PFP column has extra separation mechanisms to the exclusion of hydrophobicity. The application of PFP, for example, has been frequently reported in halogenated chemicals such as organophosphorus flame retardants (OPFRs) [35] and organophosphorus pesticides [36] because of strong steric interaction. Practically, a good separation of compounds in a complex wastewater samples was achieved by LC×LC with a combination of C18 and PFP, which enriches the versatility in developing online RPLC×RPLC methods [37].

As shown in Fig. 1 d and e, the retention of lipids from the zebrafish sample showed a broad time range for the two RPLC columns. Compared with Fig. 1 a and b, several clusters of abundant peaks were found in the chromatograms of the zebrafish extract, indicating the complexity of this sample. Despite the achievement in separation space, the coelution of some lipid groups, such as PCs, PEs, and SMs, still occurred, resulting in difficulty to distinguish isobaric lipid species (e.g., the m/z difference between PE (38:6) and PC (36:6e) is 0.0251 Da, data not shown).

The HILIC chromatograms (Fig. 1c, f) showed that the mixture of lipid standards or the lipids from the zebrafish extracts were separated based on the different polarities of the head groups. In the order of elution from the earliest to the latest, the major lipid groups in the zebrafish extract were nonpolar lipids (i.e., CER, CE, DG, and TG), PC, PE, SM, LPC and LPE, PS, and PI with increasing polarities.

Even though relatively low separation resolution was obtained by the HILIC column, it provided class-type information of lipids, which offers additional selectivity, complementary to RP chromatography.

### Comprehensive 2DLC-MS separation

#### Selectivity

The effective separation space of comprehensive 2DLC-MS will increase when increasing the orthogonality of stationary phases in the two dimensions, but the compatibility issues of different phases are critically important for the development of 2DLC systems. To make a good compromise between orthogonality and compatibility, RPLC and HILIC were finally selected for the LC×LC separation. Three different LC×LC combinations of HILIC×C18, C18×HILIC, and HILIC×PFP were compared. The C18×HILIC and HILIC×C18 were used to study the effect of column orders on the separation power of the 2DLC-MS approaches, while the C18 and PFP were used as the second dimension column (HILIC×C18 vs HILIC×PFP) to evaluate the resolution of RPLC in high solvent flow rate and their orthogonality with HILIC. The PFP×HILIC combination was excluded because the peak capacity of C18 and PFP can be easily compared based on the C18-MS and the PFP-MS results.

#### Design of the LC×LC methods

The LC×LC analysis of the zebrafish extract was optimized using the lipid standards. For the C18×HILIC combination, the individual lipid species separation of the zebrafish extract was achieved using a 100-mm-long, narrow bore (2.1 mm) C18 column equipped with 2.6-μm-core shell particles, for the first dimension (^1^D) because of universal applicability and high resolution. For the C18×HILIC, the mobile phases in the two dimensions were slightly incompatible due to a large proportion of a strong organic solvent (IPA). It is essential that the ^1^D separation of the sample should be carried out at a low flow rate (20 μL min^−1^) because small injection volumes in the second dimension will decrease a possibility of peak broadening [38, 39]. Additionally, it should fit with the two 40-μL-volume loops to collect the ^1^D effluent before the transfer to the 2D column. In order to speed up the elution of lipids in such lower flow rate, the temperature was increased to 55 °C.

For the second dimension (^2^D), a short column (50 mm) HILIC column with small particle size (1.7 μm) is important for a high-resolution separation within a short analysis time. Furthermore, the ^2^D cycle time is a key factor to minimize the effect of under-sampling which will lead to a decrease in peak capacity of the first dimension. The second dimension analysis time (sum of the gradient time and the equilibration time) is equal to the sampling time of effluent fractions from the first dimension. The ^2^D separation should be accelerated to provide an appropriate sampling frequency of the peaks transferred from the first dimension (generally 2–3 fractions per peak), while there is good separation power in the second dimension. The effect of gradient slopes in the second dimension was investigated and decreasing gradient steepness from 95 to 86.5% of organic solvent (acetonitrile) eventually provided better chromatographic resolution. The optimal conditions in the second dimension are as follows: the modulation time was set to 1 min; the flow rate of mobile phase was 2 mL min^−1^; the temperature was increased to 45 °C.

**Fig. 2:**
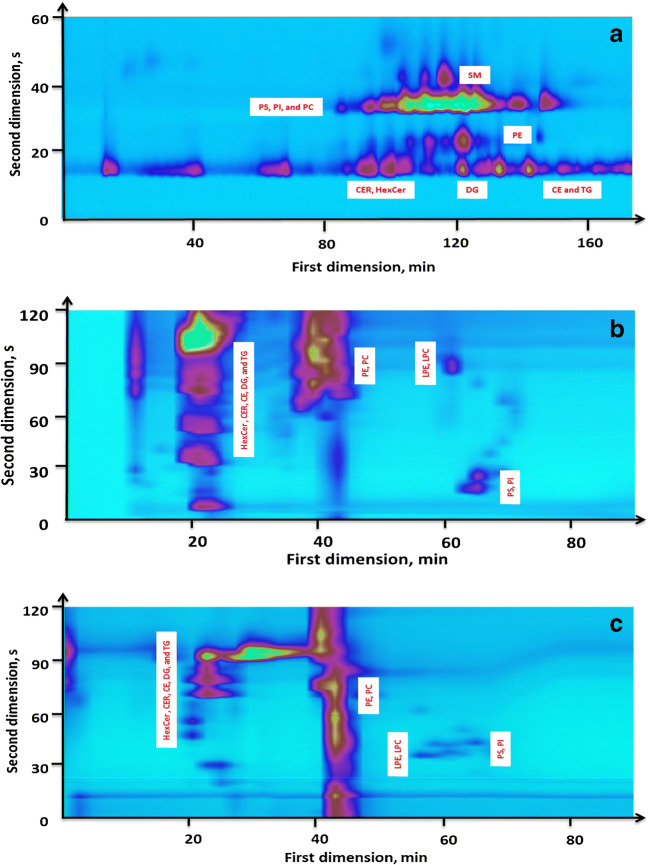
The contour plot of LC×LC chromatograms (total ion chromatograms) of the lipid extracts from pooled zebrafish embryo samples analyzed by C18×HILIC (**a**), HILIC×C18 (**b**), and HILIC×PFP (**c**) under optimal conditions. Chromatographic and MS conditions are given in the “Experimental” section

During the development of the HILIC×RPLC, class-type separation was achieved by a 150-mm HILIC column (amide-bonded stationary phase) with 3.5-μm particles. A narrower internal diameter (2.1 mm) could ensure a low ^1^D flow rate (20 μL min^−1^) which was operated at suboptimal conditions. Individual lipids that had overlapping retention times could be separated using two different RPLC columns (a C18 and a PFP columns) in the second dimension (Fig. 2b, c). In HILIC×RPLC, less peaks (data not shown) were observed compared with the C18×HILIC. To further improve the peak capacity of the 2DLC systems, instead of full gradient used above, segment gradients (shown in Table 2) were applied for the optimization of the HILIC×RPLC methods based on the large differences in hydrophobicity of the lipid species between different classes. In this application, nonpolar lipid classes eluted first (e.g., DG, TG, CER, and CE), containing both short- and long-chain fatty acids. A broad range of organic solvent, from 40 to 99%, was used in the C18 analysis, so that the lipids were better resolved. The ^2^D separation of LPC and LPE was achieved using much weaker mobile phase because of their low molecular mass. Therefore, segment gradients could offer better separation based on the fact that different solvent concentrations more closely match the retention of different lipid groups [40, 41]. In addition, it is essential to use a gradient to reduce the possibility of wrap-around of peaks, which occurs when the second dimension separation time of analytes is larger than the modulation time. Besides, it is worthy of noting that we elevated the oven temperature of the second dimension column to 55 °C, and such a high temperature resulted in the fast dispersion of analytes and the decrease of solvent viscosity, therefore, facilitating the fast elution of all lipids within a 2-min modulation time, as well as the reduction of backpressure of the instrument and column.

#### The LC×LC separation of zebrafish embryos

The contour plots of the chromatograms of the different LC×LC approaches (the C18×HILIC, the HILIC×C18, and the HILIC×PFP) showed a good separation and an excellent orthogonality (Fig. 2). Figure 2 a shows the C18×HILIC combination; similar elution orders were detected in the first dimension compared with the 1DLC-MS using a C18 column. For the second dimension separation, the retention of lipids increased, with increasing polarity from nonpolar classes (TG, DG, CER, HexCer, and CE) to the more polar lipid classes (PE and LPE, PC, PS and PI, SM and LPC). The HILIC×RPLC chromatograms are shown in Fig. 2 b and c. It was inevitable that some lipid classes partially overlap because some groups showed a broad range of retention times [42]. In the increasing order of elution, the nonpolar classes elute first in the first dimension, followed by PC and PE, SM, LPE and LPC, and PS and PI, respectively. The second dimension (RPLC) separation was based on the hydrophobicity of the lipids.

### Comparison of the 1DLC and 2DLC methods

#### Number of annotated lipid species

For comprehensive lipid profiling of zebrafish samples, the annotation and identification of lipids were performed by MS Dial (v. 3.70) based on a number of parameters (e.g., accurate m/z, isotope pattern, and retention time), with additionally the second dimension retention time (^2^*t*_R_), and confirmed by the retention time of internal isotope-labelled standards added to the samples.

In theory, the annotated lipids from a given sample should have an identical number of lipid species between the different approaches. In total, 1784 lipids belonging to 29 (sub)classes, 1059 lipid species from 27 (sub)classes, and 1123 lipids from 27 classes were tentatively found for C18×HILIC, HILIC×C18, and HILIC×PFP, respectively (Table 3). In contrast, the number of lipid species annotated was 418, 448, and 212 from the different ^1^D data sets (C18, PFP, and HILIC, respectively). The most abundant classes were TG, DG, and PC in the zebrafish embryos for all LC×LC approaches. Small differences in the number of lipid species belonging to LPC, LPE, and PE were found (Table 3). However, the majority of lipid classes, such as CER, HexCer, SHexCer, BMP, CerP, HexCer-AP, PI-Cer, MGDG, and DGDG, were found to have a much smaller number of lipids detected using one-dimensional LC than using two-dimensional LC. This is probably due to that many lipids occur at a low-ion intensity which could not be seen when they coeluted with high-concentration lipids in 1DLC. Despite cholesterol has a high concentration in biological samples, it has been reported to have lower ionization efficiency when cholesterol was detected in electrospray ionization source (ESI)-MS [43] so that it was not detected in both the HILIC and HILIC×RPLC separations probably due to an overlap of nonpolar lipids.

**Table 3:** Distribution of different lipid classes from the zebrafish embryo sample, which was detected by three conventional one-dimensional LC-MS methods and three comprehensive two-dimensional LC-MS approaches and the corresponding number of lipid species belonging to these groups

	C18	PFP	HILIC	C18×HILIC	HILIC×C18	HILIC×PFP
PC	116	114	69	170	89	143
PE	11	29	20	20	12	14
PI	12	6	2	61	12	11
PS	0	0	1	27	5	8
LPC	20	17	20	17	6	12
LPE	1	1	2	2	3	0
CL	0	0	0	2	0	9
BMP	1	2	3	59	63	35
HBMP	0	1	0	22	3	9
MG	1	1	0	0	0	0
DG	28	19	3	126	79	59
TG	100	137	29	277	164	177
MGDG	1	0	0	28	12	10
DGDG	0	0	0	32	24	16
SM	48	42	19	59	53	43
GM3	0	0	0	7	1	2
SHexCer	0	1	2	46	16	17
Cer-NS	3	2	0	38	14	15
Cer-NDS	16	14	7	173	99	161
Cer-AP	11	14	7	94	48	64
CerP	1	1	0	23	19	11
HexCer-NS	2	1	1	21	12	11
HexCer-NDS	6	5	4	157	76	68
HexCer-AP	3	1	2	55	68	35
Sphingosine	0	0	0	3	0	2
Sphinganine	1	0	0	1	1	2
Cholesterol	1	1	0	1	0	0
CE	13	11	4	48	36	51
ACar	7	6	9	27	16	12
PI-Cer	7	3	3	62	34	48
Cer-EOS	8	13	2	35	30	50
HexCer-EOS	6	6	3	91	64	30
Total lipids	418	448	212	1784	1059	1123

For the HILIC×RPLC, the separation in the first dimension is based on lipid classes, while the separation in the second dimension is based on lipid species. This is similar as was found for a stop-flow 2DLC-MS and an offline 2DLC-MS system. It has been reported by Wang et al. [28] that 372 lipids in plasma were identified by a stop-flow two-dimensional LC-MS method within 130 min, while the number of lipids identified was 284 using ^1^D RPLC analysis. Narváez-Rivas et al. [44] identified about 800 lipid species in rat plasma and rat liver by an offline two-dimensional mix-mode LC-RPLC-MS/MS method about 7 h, and approximate 400 lipids by RPLC-MS method within 31 min [17]. However, in this study, there were over 1000 lipid species annotated using the HILIC×RPLC compared with RPLC-MS of about 400 annotated lipids. This is very likely due to the loss of separation power in the first dimension where lipid species had been resolved, then re-mixed and transferred to the second dimension for further separation when using the stop-flow or offline 2DLC systems. In addition, the annotated number of lipids by the online C18×HILIC-MS was about 4 times the number of lipids detected via mass spectral matching compared with the conventional 1D RPLC-MS platform, as well as about 5 times between the HILIC×RPLC-MS and the conventional 1D HILIC-MS. The increased number of detected lipid species can be attributed mostly to the increased peak capacity provided by the second dimension in the LC×LC-MS systems. For instance, the coelution of PCs, PEs, and SMs found in ^1^D RPLC-MS was avoided by the C18×HILIC separation because the isobaric lipid species were further resolved on the basis of different polarities using HILIC in the second dimension. Correspondingly, individual lipid species could be exactly annotated after deleting the false positive data which was not in accordance with the information of m/z, the retention time in the two dimensions, and the information supported by internal standards. Overall, the performances of comprehensive two-dimensional LC methods, especially the C18×HILIC, dramatically surpass those of conventional one-dimensional LC methods.

#### Orthogonality and effective peak capacity estimation

**Fig. 3:**
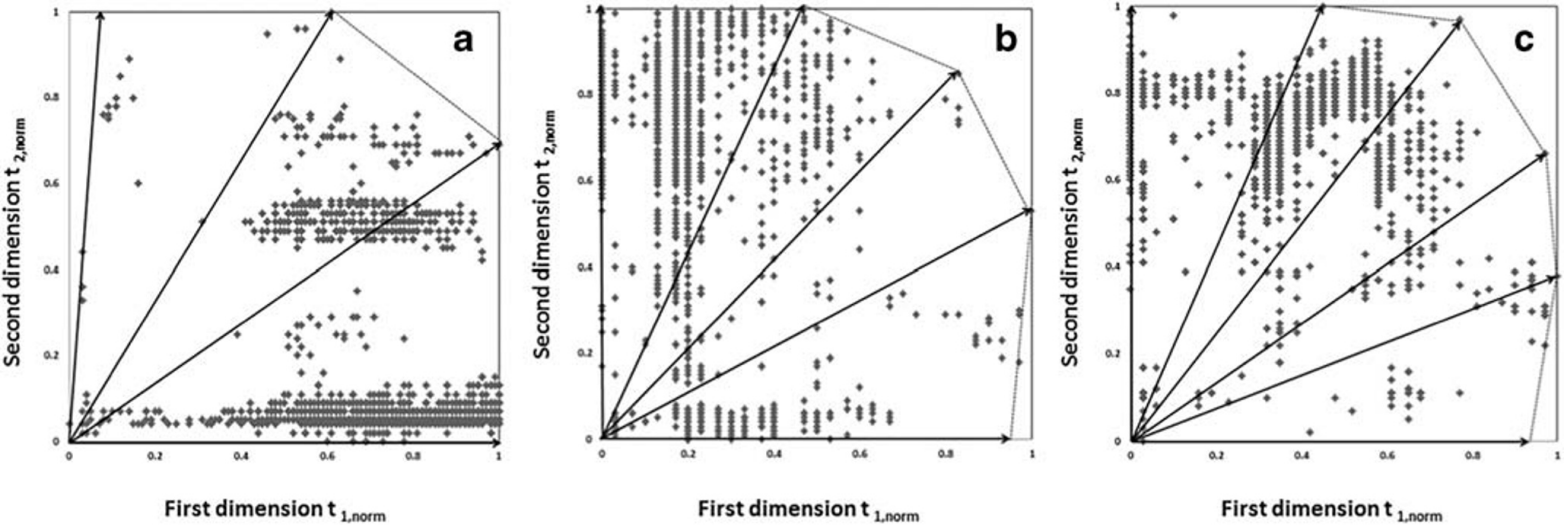
The surface coverage values of the C18×HILIC (**a**), the HILIC×C18 (**b**), and the HILIC×PFP (**c**) separations, which were estimated using all annotated lipid species from the zebrafish sample. Several vectors were introduced to simply the calculation process

In addition to the number of annotated lipids, the degree of orthogonality of the LC×LC approaches is a critical metric to evaluate a 2DLC system because there is a high correlation between the separation mechanisms in the two dimensions, resulting in a reduction in effective peak capacity of the LC×LC. Based on previous studies [45, 46], the orthogonality of comprehensive 2DLC systems was estimated by a surface coverage method (see ESM). Rather than using several lipid standards [29], all annotated lipids were included in the component pool and used to calculate the coverage metric because the size of analytes in the LC×LC separation really influences the values of orthogonality [47]. To simplify the calculation, several vectors [37] were introduced to compute the effective area of surface coverage which was, as is seen from Fig. 3, framed by black lines. The surface coverage of the LC×LC separations in the combinations of stationary phases by the C18×HILIC (a), the HILIC×C18 (b), and the HILIC×PFP (c) was 0.905, 0.907, and 0.937, respectively.

According to Eq. (2), the peak separation power of the C18×HILIC, the HILIC×C18, and HILIC×PFP separations is approximate 614, 413, and 426, respectively. The practical peak capacity of the HILIC×RPLC approaches in this study was similar to the value of 462 given by Kalili et al. [48] under similar analytical conditions. There were 143 lipid species identified by the comprehensive C18×HILIC separation with the lower peak capacity of about 369 (due to lower ^2^*n*_c_ of 4.5 vs 8 in this study) using 150-min gradient time [27].

These findings indicate that under the optimized conditions, the number of lipids annotated is positively related to the practical separation space by the LC×LC platforms, which could also be confirmed by the number of peaks in the chromatograms from individual methods (not shown).

Comparing the C18×HILIC and HILIC×C18 separations shows that the C18×HILIC method provided higher peak capacity, although the orthogonal degrees of the comprehensive 2DLC systems, where separation modes were in inverse sequence, were similar. It probably implied that under optimal conditions, the correlation between two dimensions relates closely to separation mechanisms rather than the orders of columns. Besides, different separation modes influence the peak capacities in the two dimensions. Therefore, it should be important to determine (i) the separation mechanisms in comprehensive 2DLC to study the orthogonality and (ii) the orders of selectivities. The RPLC×HILIC approaches are popular in lipidomic studies, while the HILIC×RPLC methods are widely applied for analytes which have a broad range of polarities but a short span of hydrophobic properties, such as metabolites [32], polymers [21], and therapeutic antibodies [49].

The HILIC×C18 and HILIC×PFP separations showed similar effective peak capacities. However, good orthogonality was shown for the HILIC×PFP combination, even higher than observed for the HILIC×C18 method, indicating that the HILIC coupled to PFP will be a promising platform for lipidomic studies. To further improve the peak capacity of C18-bonded columns (also C8- and C30-bonded columns) can be optimized through the steepness of gradient, mobile phase with modifiers, and column temperature. The orthogonality is unlikely to be increased because of the main separation interaction (hydrophobic properties) exists for C18 when connected to HILIC. In contrast, it is the combination of the PFP and HILIC columns that showed increasing orthogonality because of its multiple mechanisms.

In summary, though the HILIC×RPLC methods succeeded in analysis of lipid extracts from zebrafish within 100 min, the C18×HILIC method will be applied for further untargeted lipidomic studies of complex samples, from the perspective of comprehensive lipid information.

## Conclusion

As expected, the performance of comprehensive two-dimensional LC-MS greatly overtook that of the conventional 1D LC-MS in untargeted lipidomic studies of zebrafish embryo samples. The number of lipid species annotated by the conventional LC-MS methods was between 212 and 448 within 32 min, and more lipids were detected in the RPLC-MS data than the HILIC-MS data. HILIC separated the lipids based on different polarities of the head groups, which was complementary to the separation mechanisms of the C18 RPLC methods which was based on the carbon chain length and the number and positions of double bonds. The difference between a C18 and a PFP columns especially was shown for the separating of PCs and SMs.

An increase in the effective peak capacity of the C18×HILIC separation was found compared with the HILIC×C18 method, even though the orthogonal degrees of these two platforms were very similar. Correspondingly, there were more chromatographic peaks and lipids annotated by the C18×HILIC approach (1784 vs 1059 lipid species than by the HILIC×C18 method). Comparison of the HILIC×C18 and HILIC×PFP showed a better orthogonality of the latter technique which indicated that the HILIC coupled to PFP had great potential for untargeted lipidomics.

Overall, within 100 min, the HILIC×RPLC methods provided a good class-type separation under the fact that the number of annotated lipids by 2DLC systems was about 2.5 times and 5 times the number of 1D RPLC-MS and HILIC-MS, respectively. However, for comprehensive lipid profiling, the C18×HILIC was selected to be applied for further lipidomics studies of complex zebrafish samples.

## Electronic supplementary material

ESM 1 (PDF 336 kb)
