# Supplementary material for: Evaluation of LC-MS and LC×LC-MS in analysis of zebrafish embryo samples for comprehensive lipid profiling
Source: Anal Bioanal Chem. 2020 Apr 29;412(18):4313–25. doi: 10.1007/s00216-020-02661-1 (PMC7320064; doi:10.1007/s00216-020-02661-1)
Supplement: Supplementary file 1 — (PDF 336 kb) [file 216_2020_2661_MOESM1_ESM.pdf]

Analytical and Bioanalytical Chemistry

Electronic Supplementary Material

Evaluation of LC-MS and LC×LC-MS in analysis of zebrafish embryo samples for comprehensive lipid profiling

Mengmeng Xu, Jessica Legradi, Pim Leonards

Additional files available under 10.1007/s00216-020-02661-1.

Tables S1 to S4 see separate Excel files.

Table S5 Detailed information of internal-labelled lipid standards added to the lipid extracts from zebrafish samples separated by one and two-dimensional LC-MS methods under the optimal conditions and used to provide quality control for annotation and identification of lipid species in zebrafish samples

Lipid class	m/z	adduct
15:0-18:1(d7) PC	753.6136	$[M+H]^+$
15:0-18:1(d7) PE	711.5640	$[M+H]^+$
15:0-18:1(d7) PS	755.5472	$[M+H]^+$
15:0-18:1(d7) PG	740.5550	$[M+H]^+$
15:0-18:1(d7) PI	828.5410	$[M+H]^+$
18:1(d7) LPC	529.3987	$[M+H]^+$
18:1(d7) LPE	487.3517	$[M+H]^+$
18:1(d7) Chol Ester	675.6693	$[M+NH_4]^+$
15:0-18:1(d7) DG	605.5804	$[M+NH_4]^+$
15:0-18:1(d7)-15:0 TG	829.7928	$[M+NH_4]^+$
18:1(d9) SM	738.6472	$[M+H]^+$
Cholesterol (d7)	411.3881	$[M-H_2O+H]^+$

The orthogonality of the LC×LC methods was calculated by surface coverage using lipid extracts from pool samples

The orthogonality of the LC×LC systems was estimated using real samples instead of a mixture of lipid standards because the size of samples really affects the validation of orthogonality evaluation. In this study, the surface coverage is selected as an orthogonal estimation metric according to previous studies and the procedure is as followed:

First, the retention time of the first dimension (^1t_R) and second dimension (^2t_R) of all the peaks annotated were normalized using Eq. (1):

$$RT_{,norm(i)} = \frac{RT_{(i)} - RT_{,first}}{RT_{,last} - RT_{,first}} \quad (1)$$

In this equation, $RT_{(i)}$ is the retention time of the peak i in sequence, and $RT_{,\ first}$ and $RT_{,\ last}$ represent the retention time of the peaks eluted in first and last place, respectively.

After normalization, a range in retention times of all the peaks is between 0 and 1. According to study of Ouyang et al. (2015), we used several vectors to simplify the calculation of effective separation space which is equal to the area of irregular graphics framed by black lines. Therefore, the areas of graphics were calculated by totally summing the product of the vector cross demonstrated in Eq. (2):

$$f(coverage) = \frac{1}{2}\left|\vec{a} \times \vec{b}\right| + \frac{1}{2}\left|\vec{b} \times \vec{c}\right| + \frac{1}{2}\left|\vec{c} \times \vec{d}\right| + \cdots \quad (2)$$

Finally, the value of f(coverage) is regarded as the metric of orthogonality. Under the condition that two dimensions were absolutely uncorrelated, the surface coverage is 1. On the contrary, if the separation was far from orthogonal, the value of the metric is almost 0.

Table S6 Distribution of the relative standard deviation (RSD) of the retention time (t_R) for the C18-MS and the HILIC-MS methods (n=5) and the relative standard deviation of both the first dimension retention time (^1t_R) and the second dimension retention time for the C18×HILIC data (n=5), which were performed by lipid standards. The chromatographic and MS conditions were present in the Experimental section

Lipid Class	RSD[1] (%)	RSD[2] (%)	^1t_R RSD[3] (%)	^2t_R RSD[3] (%)
TG	0.43	0.46	0.43	7.99
DG	0.84	4.01	2.38	4.67
PC	1.07	0.33	2.02	2.83
PE	0.93	0.28	2.88	6.03
PS	0.85	2.84	2.17	3.31
PI	19.94	0.23	2.11	0.65
LPC	2.02	0.22	8.66	1.51
LPE	0.88	0.20	28.28	3.98
CE	0.21	1.12	1.99	5.68
SM	1.18	0.17	2.03	1.64
CER	0.47	9.07	1.87	7.02

1: the C18-MS method;

2: the HILIC-MS method;

3: the C18×HILIC method

Table S7 The abbreviations of all lipid classes

Lipid class	Abbreviation
Acylcarnitine	ACar
Cholesteryl ester	CE
Monoacylglycerol	MG
Diacylglycerol	DG
Triacylglycerol	TG
Monogalactosyldiacylglycerol	MGDG
Digalactosyldiacylglycerol	DGDG
Cholesterol	Cholesterol
Lysophophatidylcholine	LPC
Lysophosphatidylethanolamine	LPE
Phosphatidylcholine	PC
Phosphatidylethanolamine	PE
Phosphatidylinositol	PI
Phosphatidylserine	PS
Bismonoacylglycerophosphate	BMP
Hemibismonoacylglycerophosphate	HBMP
Cardiolipin	CL
Sphingomyelin	SM
Sulfatide	SHexCer
Ganglioside GM3	GM3

Ceramide non-hydroxyfatty acid-sphingosine	Cer-NS
Ceramide non-hydroxyfatty acid-dihydrosphingosine	Cer-NDS
Ceramide Esterified omega-hydroxy fatty acid-sphingosine	Cer-EOS
Ceramide alpha-hydroxy fatty acid-phytospingosine	Cer-AP
Ceramide 1-phosphates	CerP
Hexosylceramide non-hydroxyfatty acid-sphingosine	HexCer-NS
Hexosylceramide non-hydroxyfatty acid-dihydrosphingosine	HexCer-NDS
Hexosylceramide Esterified omega-hydroxy fatty acid-sphingosine	HexCer-EOS
Hexosylceramide alpha-hydroxy fatty acid-phytospingosine	HexCer-AP
Sphingosine	Sphingosine
Sphinganine	Sphinganine
Phytosphingosine	Phytosphingosine
